# Data extracted from olive oil mill waste exposed to ambient conditions

**DOI:** 10.1016/j.dib.2019.104555

**Published:** 2019-09-23

**Authors:** Luis González-Martínez, D. Hernández, César A. Astudillo, Fabián Silva A, David Gabriel

**Affiliations:** a Instituto de Química de Recursos Naturales, Universidad de Talca, Talca, Chile; b Departamento de Ciencias de la Computación, Universidad de Talca, Curicó, Chile; c GENOCOV Research Group, Department of Chemical, Biological and Environmental Engineering, Universitat Autònoma de Barcelona, 08193, Bellaterra, Spain

**Keywords:** Olive oil mill waste, Open-air reservoirs, Odorants, Ilumina sequencing

## Abstract

Recent studies show that the process of extraction of olive oil results in a large amount of waste. Around 20% the oil is obtained in the process and the remaining 80% corresponds to mainly two types of waste, known as orujo and alperujo. These residues were stored in pools for 6 months in an uncontrolled environment. The reservoirs are open and generate Odorous Volatile Organic Compounds (VOCs) as products of waste decomposition. The data in this article corresponds of physical-chemical compounds of olive oil mill waste exposed to ambient conditions. The data was obtained from two different oil mills, namely, Almazara del Pacífico located in the Alto Pangue area, Talca, Chile; and Agricola y Forestal Don Rafael oil mill, Molina, Chile. Samples were extracted directly from the oil mills to fill 200 L plastic containers that simulated the waste storage in oil mill reservoirs. Each sample was identified and standardized to a mass of 150 kg and moved and stored under uncontrolled ambient conditions at the Universidad de Talca, Curicó, Chile.

**Table:** Specifications Table

Subject	Environmental Chemistry
Specific subject area	Odorous Volatile Organic Compounds.
Type of data	Table
How data were acquired	Chemical sampling from pools in open reservoir with Gas Chromatograph with Mass Spectrometer (GC/MS) (Thermo Fisher Scientific, Trace 1300/ISQELT)
Data format	Raw
Parameters for data collection	The containers were exposed to climatic conditions to simulate the conditions of the real problem.
Description of data collection	For the initial samples of fresh wastes from oil mills, a homogeneous sample of 1 kg was taken from each container and mixed to form a single sample per type of fresh waste. The samples were analyzed in triplicate to determine the initial characteristics of each residue. For monthly samples, eight sub-samples of 250 g each were obtained.
Data source location	Institution: Almazara del Pacífico and Agrícola y Forestal Don Rafael oil mill City/Town/Region: Alto Pangue area, Talca and Molina, respectively. Country: Chile
Data accessibility	Direct URL to data: goo.gl/tnPmVE
Related research article	Authors: Hernández D, Astudillo CA, Fernández-Palacios E, Cataldo F, Tenreiro C, Gabriel D Title: Evolution of physical-chemical parameters, microbial diversity and VOCemissions of olive oil mill waste exposed to ambient conditions in openreservoirs. Journal: Waste Management https://doi.org/10.1016/j.wasman.2018.08.022

**Table:** 

**Value of the data** • The data provides important information about the physical-chemical process of waste decomposition during the extraction of olive oil. • The data is useful for predicting the relationship between atmospheric variables and the variation of the physical-chemical properties of the oil mill waste. • The data shows how the waste decomposition over time is a result of the decomposition of the organic matter due to the biological and chemical action. • The data can be used to counteract or reuse waste. • Some of the data was analyzed in a previous paper [1]. The data reported in the present study corresponds to all the collected information. This information can be useful for more detailed studies. • To facilitate the analysis, the data has been placed in a spreadsheet which is publicly available.

## 1 Data

The data in the article specified in Ref. [1], provides a comprehensive insight into the evolution of physical-chemical parameters of olive oil mill waste exposed to ambient condition in open reservoirs. The Measurements were made for six months, from June to November, involving environmental and composite indicators. These measurements were extracted from two distinct locations: Alto Pangue, Talca, Chile; and Molina, Chile. The study considered two different types of waste, namely Orujo and Alperujo. The Table 1 shows all the parameters and their respective unit measures [1]. The parameters were separated into two subsets. The first subset of parameters is composed of environmental variables such as precipitation, minimum temperature, maximum temperature, wind speed, and environmental humidity. The second subset considered composite indicators such as content moisture, crude protein, measuring heating value, ashes, crude fiber, fats, pH, molds and yeasts, and total phenolic, all these parameters are described with their measurements in the Table 1.

**Table 1:** Parameters under study as well as their respective unit of measurement.

Parameter	Measurement
Precipitation	Millimeter (mm)
Minimum temperature	Celsius degree (°C)
Maximum temperature	Celsius degree (°C)
Wind Speed	Kilometer Per Hour (Km/h)
Relative Humidity	Percentage (%)
Moisture Content	Percentage (%)
Crude Protein	Percentage (%)
Measuring heating value	Mega joules (MJ)
Ashes	Percentage (%)
Crude Fiber	Percentage (%)
Fats	Percentage (%)
pH	Dimensionless quantity
Molds and yeasts	(UFC g-1)
Total Phenolic	(mg-L)

## 2 Experimental design, materials, and methods

**Fig. 1:**
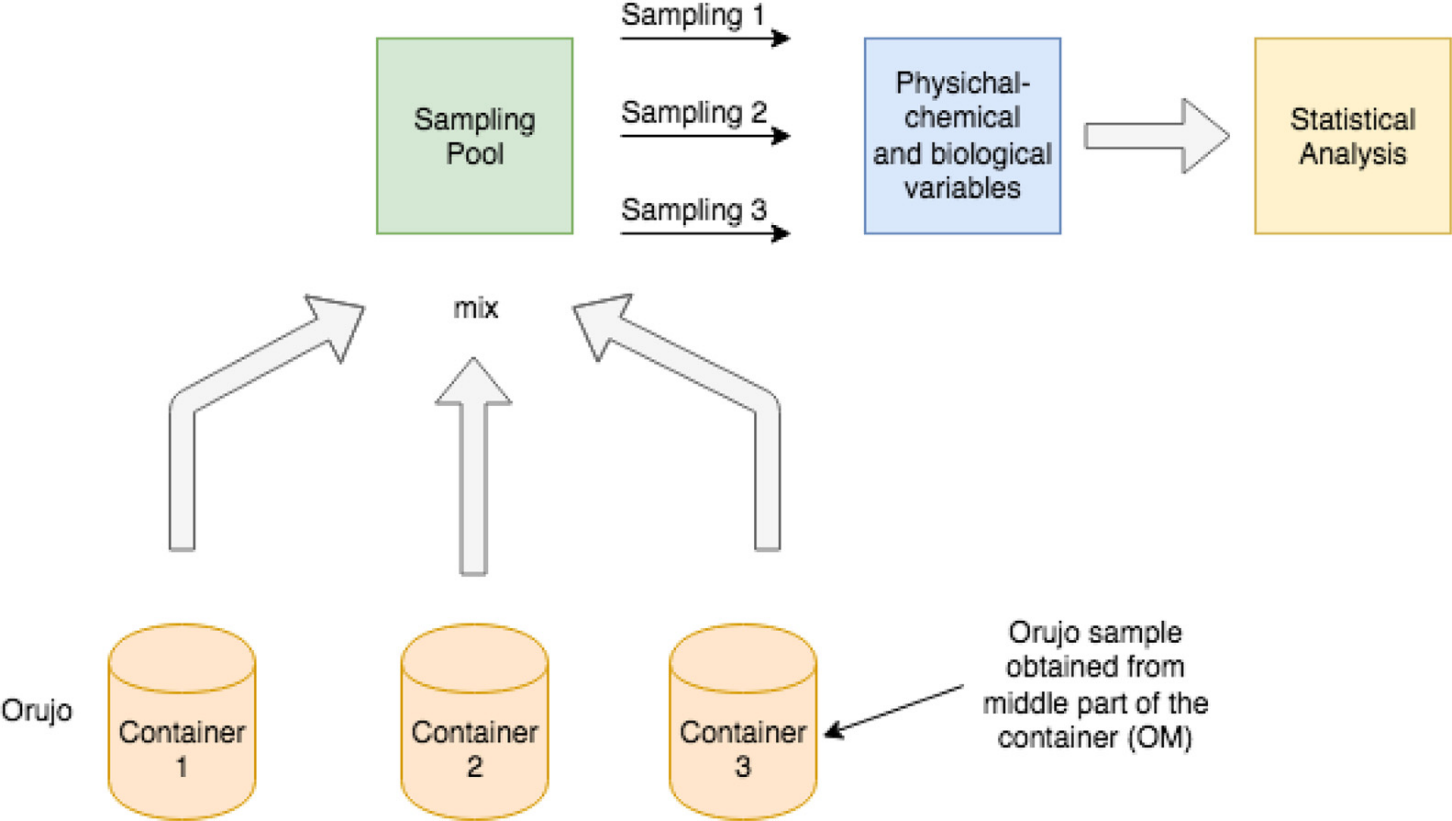
Orujo sampling obtained from the middle part of the container.

The sampling method is depicted in Fig. 1. The diagram shows the case when obtaining the orujo from the middle part of the container. The other three waste sampling processes, i.e., orujo obtained from the top (OT), as well as alperujo obtained from the top (AT) and middle part of the container (AM), are analogous.

Fig. 1 shows three containers that store the mixed waste extracted from the two oil mills. Each container was stored under uncontrolled ambient conditions and were kept open and outdoors between June and November. Each month, samples were extracted with the dredge and mixed to obtain a single representative sample. From this pool three sub-samples were obtained, and the physical-chemical variables detailed in Table 1 were measured. At the same time, sub-samples of the intermediate part (half of the height and diameter of each reservoir) were mixed to obtain a single representative sample.

The resultant data is detailed in Table 3. The data is divided in four groups, and include the measurements for the alperujo obtained from the top of the container (AT), alperujo obtained from the middle part of the container (AM), orujo obtained from the top of the container (oT), and orujo obtained from the middle part of the container (OM). For each group, the measurements indicated in Table 2 are shown for a period of six months. In each case, the average values and standard error are reported.

**Table 2:** Environmental indicators acquired.

Parameter	June	July	August	September	October	November
Precipitation (mm)	10.60	25.60	4.50	3.20	15.10	2.50
Minimum Temperature (Tmin) (°C)	4.50	5.20	6.00	5.60	8.30	9.80
Maximum Temperature (Tmax) (°C)	13.00	14.20	19.00	21.50	22.50	25.20
Wind Speed (Wind) (Km $hˆ-1$)	4.00	10.40	7.50	8.50	9.20	10.50
Relative Humidity (%)	86.00	86.00	81.00	77.00	71.00	58.00

**Table 3:** Evolution of physical-chemical parameters of A and O in the upper (AT and AO) and in the intermediate (AM and OM) part of the containers.

Parameter	June	July	August	September	October	November	Average
AT							
Moisture (%)	56.9 ± 0.1	62.7 ± 0.9	57.7 ± 1.2	43.0 ± 0.1	37.9 ± 1.0	32.9 ± 1.0	48.5 ± 12.2
Proteins (%)	8.5 ± 1.1	8.6 ± 0.2	8.2 ± 1.1	7.5 ± 0.7	6.4 ± 0.9	6.0 ± 0.9	7.5 ± 1.1
Measuring heating value (MJ)	22.5 ± 0.6	22.9 ± 0.8	22.9 ± 1.0	22.9 ± 1.0	22.7 ± 1.4	22.3 ± 1.3	22.7 ± 0.3
Ashes (%)	1.9 ± 0.2	1.8 ± 0.1	1.7 ± 0.1	1.9 ± 0.1	2.1 ± 0.1	2.3 ± 0.2	1.9 ± 0.2
Fibres (%)	91.5 ± 0.6	90.8 ± 0.4	91.0 ± 1.0	89.6 ± 0.2	88.7 ± 1.2	88.5 ± 1.1	90.0 ± 1.3
Fats (%)	12.0 ± 0.9	11.0 ± 1.0	10.4 ± 1.6	9.5 ± 1.1	88.7 ± 0.6	8.2 ± 1.2	10.0 ± 1.4
Ph	5.7 ± 0.3	5.6 ± 0.1	5.6 ± 0.1	5.6 ± 0.1	5.7 ± 0.2	5.7 ± 0.2	5.6 ± 0.0
Molds and yeasts (UFC g-1)	4.0 ± 0.0	5.0 ± 1.0	4.0 ± 0.0	6.0 ± 1.0	7.0 ± 6.0	6.0 ± 0.0	5.3 ± 1.2
Total Phenolic (mg-L)	13.1 ± 0.4	8.8 ± 0.2	8.6 ± 0.4	8.4 ± 0.5	12.4 ± 0.5	12.3 ± 0.5	10.6 ± 2.2
AM							
Moisture (%)	60.4 ± 1.3	68.9 ± 1.0	60.8 ± 1.6	56.0 ± 0.1	47.8 ± 1.5	43.0 ± 1.5	56.1 ± 9.4
Proteins (%)	8.3 ± 0.6	8.2 ± 0.7	8.1 ± 0.8	7.0 ± 0.6	6.9 ± 0.2	6.9 ± 0.5	7.5 ± 0.8
Measuring heating value (MJ)	22.5 ± 0.1	22.3 ± 0.1	23.0 ± 1.2	22.7 ± 1.4	22.3 ± 0.1	22.3 ± 0.9	22.6 ± 0.3
Ashes (%)	1.9 ± 0.1	1.3 ± 0.1	1.3 ± 0.1	1.9 ± 0.2	2.1 ± 0.2	2.2 ± 0.3	1.8 ± 0.4
Fibres (%)	88.5 ± 0.5	89.8 ± 0.7	90.3 ± 0.6	89.6 ± 0.2	88.9 ± 0.5	88.2 ± 0.7	89.2 ± 0.8
Fats (%)	8.5 ± 1.0	8.6 ± 1.0	8.6 ± 0.9	8.3 ± 1.1	8.0 ± 1.0	7.8 ± 1.1	8.3 ± 0.3
Ph	5.7 ± 0.3	5.6 ± 0.1	5.6 ± 0.1	5.7 ± 0.0	5.6 ± 0.2	5.7 ± 0.2	5.7 ± 0.0
Molds and yeasts (UFC g-1)	4.0 ± 0.0	5.0 ± 0.0	2.0 ± 1.0	3.0 ± 0.0	5.0 ± 0.0	1.0 ± 0.0	3.3 ± 1.6
Total Phenolic (mg-L)	13.3 ± 0.5	8.6 ± 0.5	8.9 ± 0.4	8.5 ± 0.9	12.3 ± 0.5	12.4 ± 0.6	10.7 ± 2.2
OT							
Moisture (%)	69.9 ± 0.1	70.7 ± 1.3	72.7 ± 0.3	69.0 ± 1.2	56.9 ± 0.5	41.5 ± 1.5	63.4 ± 12.1
Proteins (%)	8.4 ± 0.7	8.0 ± 1.0	7.6 ± 0.9	7.1 ± 0.2	6.3 ± 0.2	5.8 ± 0.1	7.2 ± 1.0
Measuring heating value (MJ)	22.1 ± 1.0	22.2 ± 1.1	22.3 ± 1.3	22.6 ± 1.4	22.9 ± 1.0	23.0 ± 1.2	22.5 ± 0.4
Ashes (%)	2.9 ± 0.3	3.2 ± 0.1	3.5 ± 0.2	3.7 ± 0.1	3.7 ± 0.1	4.3 ± 0.2	3.6 ± 0.5
Fibres (%)	87.5 ± 0.9	88.8 ± 2.2	85.2 ± 1.3	80.5 ± 1.7	78.5 ± 1.6	83.3 ± 1.0	83.9 ± 4.0
Fats (%)	11.4 ± 1.4	11.3 ± 1.5	10.8 ± 0.7	10.5 ± 1.3	10.0 ± 0.1	9.6 ± 1.4	10.6 ± 0.7
Ph	5.8 ± 0.2	5.8 ± 0.2	5.7 ± 0.1	5.6 ± 0.1	5.7 ± 0.2	5.6 ± 0.1	5.7 ± 0.1
Molds and yeasts (UFC g-1)	5.0 ± 0.0	7.0 ± 0.0	4.0 ± 0.0	4.0 ± 0.0	5.0 ± 0.0	7.0 ± 1.0	5.3 ± 1.4
Total Phenolic (mg-L)	13.6 ± 0.6	13.4 ± 0.6	13.6 ± 0.4	16.4 ± 0.5	18.4 ± 0.8	22.3 ± 0.6	16.3 ± 3.6
OM							
Moisture (%)	68.4 ± 0.1	70.7 ± 1.3	73.8 ± 0.5	68.0 ± 1.3	57.4 ± 1.6	47.3 ± 1.0	64.6 ± 10.3
Proteins (%)	8.3 ± 0.7	8.0 ± 1.0	7.9 ± 0.1	7.0 ± 0.4	6.5 ± 0.4	6.1 ± 0.6	7.3 ± 0.9
Measuring heating value (MJ)	21.9 ± 1.0	22.2 ± 1.1	22.1 ± 1.0	22.2 ± 1.1	22.7 ± 1.4	22.6 ± 0.5	22.5 ± 0.6
Ashes (%)	3.5 ± 0.3	3.2 ± 0.1	3.7 ± 0.2	4.0 ± 0.2	4.5 ± 0.3	5.0 ± 0.2	4.0 ± 0.6
Fibres (%)	84.3 ± 0.9	88.8 ± 2.2	87.0 ± 1.0	84.2 ± 1.6	80.3 ± 1.9	81.6 ± 1.2	83.9 ± 2.5
Fats (%)	8.33 ± 1.4	11.3 ± 1.5	7.3 ± 0.9	6.6 ± 0.9	6.0 ± 1.0	5.6 ± 1.9	18.6 ± 1.0
Ph	5.8 ± 0.2	5.8 ± 0.2	5.8 ± 0.1	5.8 ± 0.2	5.7 ± 0.2	5.7 ± 0.2	5.7 ± 0.1
Molds and yeasts (UFC g-1)	2.0 ± 0.0	5.0 ± 0.0	2.0 ± 1.0	4.0 ± 0.0	3.0 ± 0.0	2.0 ± 0.0	3.0 ± 1.3
Total Phenolic (mg-L)	14.6 ± 0.6	13.4 ± 0.6	13.8 ± 0.3	15.2 ± 0.1	19.2 ± 1.1	21.2 ± 0.9	16.1 ± 3.4
